# Epidemiological characteristics of skiing injuries: a retrospective study based on the 2024–2025 Ski season

**DOI:** 10.3389/fspor.2026.1849568

**Published:** 2026-05-15

**Authors:** Shi Xinqi, Liu Dawei, Jiang Wei, Shen Jianyu, Yang Yuping

**Affiliations:** 1 Department of Sports Medicine, Beijing Key Laboratory of Sports Injuries of Joints, Peking University Third Hospital, Institute of Sports Medicine of Peking University, Beijing, China; 2 Department of Sports Medicine, Chongli Campus, Peking University Third Hospital, Zhangjiakou, Hebei Province, China

**Keywords:** epidemiological characteristics, gender differences, injury prevention, skiing injuries, snow sports

## Abstract

**Background:**

This study analyzed patients treated for skiing injuries at Chongli Campus, Peking University Third Hospital during the 2024–2025 ski season to clarify the epidemiological patterns and associated factors of skiing injuries, and to provide data support for developing preventive measures.

**Methods:**

A retrospective study design was adopted. Clinical data from 4,270 patients with skiing injuries treated between November 2024 and April 2025 were collected. Chi-square tests (with Cramér's V effect sizes) and multinomial logistic regression were used for statistical analysis.

**Results:**

Injury location were significantly associated with gender, annual skiing expenditure, skiing equipment, skiing experience and skill level (all *P* < 0.001). Low-consumption skiers were mainly beginners with <2 years of experience and predominantly sustained lower extremity injuries, whereas high-consumption skiers were mostly expert-level skiers with >5 years of experience and had a higher prevalence of upper extremity injuries. Multinomial logistic regression showed that age, male gender (OR for head/neck vs. upper extremity = 0.77, 95% CI: 0.63–0.94), low consumption (OR for head/neck = 1.79, 95% CI: 1.14–2.80), and beginner skill level (OR for lower extremity vs. upper extremity = 4.58, 95% CI: 2.13–9.81) were independent predictors of injury location.

**Conclusions:**

Differentiated and individualized preventive measures should be implemented for different groups of skiers to reduce the occurrence of skiing injuries and promote the healthy development of ice and snow sports.

## 1 Introduction

Skiing is one of the most popular winter sports worldwide, and its popularity has surged in China since the successful hosting of the 2022 Beijing Winter Olympic and Paralympic Games, evolving from a niche sport to a mainstream recreational activity for the general public. However, skiing is a high-risk sport that requires excellent balance, coordination, and the ability to respond to sudden on-slope situations, which inevitably leads to a relatively high incidence of skiing-related injuries. Previous research has documented 753 sports injuries recorded over an entire ski season at the Wanlong Ski Resort, with a skiing injury incidence rate of 1.98 per 1,000 skiing days and 60.2% of all injuries attributed to skiing activities (1). Moreover, the injury rate is even higher among professional skiers. Another study reported 2,644 skiing injuries occurring in a single month (February) across 47 ski resorts in Japan, reflecting the global prevalence of skiing injury risks (2).

As the host venue for the skiing events of the 2022 Beijing Winter Olympics and Paralympics, Chongli District has emerged as a premier skiing destination in northern China, with a rapid increase in ski tourism volume. The district received 4.4232 million tourist visits during the 2023–2024 ski season and a further 5.2346 million visits in the 2024–2025 ski season, representing an 18% year-on-year growth. The booming ski tourism industry in Chongli has highlighted the urgent need to investigate the epidemiological characteristics of skiing injuries in this region and develop scientific prevention and control measures.

Despite the growing popularity of skiing in China, there remains a paucity of localized epidemiological studies on skiing injuries, resulting in limited evidence-based guidance and prevention strategies for skiers and ski resort management. To address this research gap, and enhance public awareness of snow sports injury prevention, this study conducted a retrospective analysis of 4,270 patients treated for skiing injuries at the Chongli Campus of Peking University Third Hospital during the 2024–2025 ski season. By exploring the characteristics of injury locations and their associated risk factors, this study aims to reveal the occurrence patterns of skiing injuries in the Chongli skiing cluster and provide robust data support for formulating effective injury prevention measures and improving medical security for snow sports in China.

## 2 Materials and methods

### 2.1 General information

This retrospective study was conducted at the Chongli Campus of Peking University Third Hospital, a national regional medical center for sports trauma in Hebei Province. The campus is geographically close to numerous ski resorts in Chongli District, with the nearest resort only 5.5 km away and a maximum driving time of less than 25 min to all surrounding resorts, making it the primary medical institution for treating skiing injuries in the region. The campus has well-established Orthopedics and Sports Medicine Departments, a comprehensive skiing injury registration information system, and provides full medical support for snow sports activities in the area.

Clinical data of patients treated for skiing injuries at the campus from November 2024 to March 2025 were collected, including a total of 4,270 injury cases. The data were retrieved from the Fangzheng International Electronic Medical Record System V1.0, a dedicated skiing injury registration system that records detailed patient information: gender, age, injury date and time, injury cause and type, specific injury location, and injury severity. Among the 4,270 cases, 2,192 (51.33%) were male and 2,078 (48.67%) were female. The study involved retrospective analysis of anonymized clinical data from human participants. Because this was a retrospective anonymous epidemiological study, the requirement for informed consent was waived by the ethics committee. Patient confidentiality was protected by de-identifying all data before analysis.

### 2.2 Methods

#### 2.2.1 Injury classification

Injuries were initially classified into four anatomical regions based on the affected body part: head and neck, trunk, upper extremities, and lower extremities. For standardized classification, sternoclavicular joint injuries were categorized as trunk injuries, acromioclavicular joint injuries as upper extremity injuries, hip injuries as lower extremity injuries, and gluteal injuries as trunk injuries. Patients with multiple-site injuries were recorded separately for each injured region (3).

#### 2.2.2 Categorization of study variables

For statistical analysis, relevant variables were categorized based on common epidemiological research practices and industry standards.

##### 2.2.2.1 Education level

Divided into four levels (primary school or below, junior high school, senior high school, bachelor’s degree or above) in accordance with the conventions of China’s National Physical Fitness Monitoring and sports population statistics.

##### 2.2.2.2 Annual skiing expenditure

Measured as the total economic investment in skiing for the 2024–2025 season, divided into four intervals (< 1000 RMB, 1000-2000 RMB, 2000-3000 RMB, > 3000 RMB). Annual skiing expenditure reflects financial commitment.

##### 2.2.2.3 Skiing experience

Defined as the number of consecutive years of regular skiing participation, rounded to the nearest whole year.

##### 2.2.2.4 Self-reported skill level

Classified into three levels (novice/beginner, intermediate, expert/advanced) by integrating the China Ski Association Skill Level Standards and the skill development stage theory in sports training. Self-reported skill level may introduce classification bias, as it lacks objective validation.

##### 2.2.2.5 Preferred ski trail type

Divided into beginner, intermediate, and advanced trails based on slope gradient, difficulty, and International Ski Federation (FIS) classification criteria.

##### 2.2.2.6 Ski equipment

Recorded as snowboard or alpine skis (two boards). Preferred skiing discipline: Categorized into freestyle skiing, recreational skiing, and backcountry skiing (natural terrain challenge).

##### 2.2.2.7 Injury mechanism

Classified into three main types based on epidemiological investigation methods: self-fall, active collision, and being collided with.

##### 2.2.2.8 Protective gear use

Recorded as the actual wearing status of key protective gear (helmet, knee pads, hip pads, wrist guards, etc.) during skiing, used as an indicator to evaluate risk avoidance behavior and sports safety awareness.

#### 2.2.3 Classification of injury prevention strategies

Based on the analysis of injury types and associated risk factors, skiing injury prevention strategies were classified into three categories in line with sports medicine epidemiological guidelines (4).

##### 2.2.3.1 Universal prevention

Applicable to all skiing participants, including basic safety education, standard ski trail use, and mandatory basic protective gear wear.

##### 2.2.3.2 Selective prevention

Targeted prevention recommendations based on demographic characteristics (age, gender) and injury type, such as targeted strength training for high-risk groups.

##### 2.2.3.3 Indicated prevention

Individualized intervention and rehabilitation measures for skiers with identified individual risk factors (e.g., poor skiing posture, inadequate muscle strength, history of previous injuries).

### 2.3 Statistical analysis

Descriptive statistics (case number, gender ratio, age distribution, injury location frequency) were analyzed using Microsoft Excel. For the analysis of injury causes and specific injury location correlations with other variables, IBM SPSS Statistics 27.0.1 was used. Categorical variables were presented as frequency (n) and percentage (%). The chi-square (*χ*^2^) test was applied for intergroup comparisons of categorical data. For datasets with missing values, only available cases were included in the analysis to determine intergroup differences. To identify independent predictors of injury location while controlling for potential confounders, multinomial logistic regression was performed with injury location (upper extremity as reference) as the dependent variable and age, gender, annual consumption, skiing experience, skill level, and equipment type as independent variables. Adjusted odds ratios (OR) with 95% confidence intervals (CI) are reported. A two-tailed *P*-value < 0.05 was considered statistically significant for all analyses.

## 3 Results

### 3.1 Study population and overall distribution of injuries

A total of 4,270 skiing injury cases were included in this study, with patients aged 2–86 years and a mean age of 29.12 ± 11.43 years. The age distribution was significantly skewed towards young and middle-aged adults with 2,510 cases (58.78%) occurring in the 20–35 age group.

In terms of injury location, upper extremity injuries were the most frequent (1,487 cases, 34.82%) followed by lower extremity injuries (1,404 cases, 32.88%) trunk injuries (724 cases, 16.95%) and head and neck injuries (655 cases, 15.35%). Chi-square test confirmed a highly significant correlation between injury location and gender (*P* < 0.001, Table 1). Male skiers had a higher proportion of upper extremity injuries (848 cases, 38.69% of male injuries), while female skiers were predominantly affected by lower extremity injuries (784 cases, 37.72% of female injuries). Cramér's V for gender-injury location association = 0.115.

**Table 1:** Correlation between gender and injury location (*n*).

Gender	Injury Location					*χ* ^2^	*df*	*P*-value
	Upper Extremities	Lower Extremities	Trunk	Head and Neck	Total			
Male	848	620	405	319	2192			
Female	639	784	319	336	2078			
Total	1487	1404	724	655	4270	56.19	3	<0.001

df, degrees of freedom.

### 3.2 Correlation analysis between injury location and multivariate variables

Chi-square test was used to analyze the association between injury location and five key variables: gender, annual skiing expenditure, skiing equipment, skiing experience, and skill level. The results showed that all variables were significantly associated with injury location (all *P* < 0.001, Table 2). Effect sizes (Cramér's V) ranged from 0.107 to 0.224, indicating small-to-moderate associations. The top 3 variables in terms of correlation strength were: skiing experience (*χ*^2^ = 428.6, df = 6, V = 0.224), annual consumption (*χ*^2^ = 372.8, df = 9, V = 0.171), and skill level (*χ*^2^ = 345.7, df = 6, V = 0.201).

**Table 2:** Results of correlation analysis between injury location and multivariate variables.

Variable	χ^2^	*df*	*P*-value	Cramér's V
Gender	56.19	3	<0.001	0.115
Annual skiing expenditure	372.8	9	<0.001	0.171
Skiing equipment	49.3	3	<0.001	0.107
Skiing experience	428.6	6	<0.001	0.224
Self-reported skill level	345.7	6	<0.001	0.201

df, degrees of freedom.

Further subgroup analysis revealed a significant correlation between gender and annual skiing expenditure as well (*P* < 0.001, Table 3). Specific quantitative data showed that 57% of male skiers had a annual skiing expenditure exceeding 3000 RMB, while the expenditure level of most female skiers was below 2000 RMB.

**Table 3:** Correlation between gender and annual skiing expenditure.

Variable	χ^2^	*df*	*P*-value
Annual skiing expenditure	37.63	11	<0.001

df, degrees of freedom.

### 3.3 Analysis of injury characteristics based on skiing experience

There were significant differences in skiing equipment selection, skill level and injury location across different skiing experience stages (all *P* < 0.001, Table 4). For skiers with <2 years of experience, 58.08% used double boards, and 78.6% skied on beginner slopes. In contrast, 67.3% of those with >5 years of experience used double boards and preferred advanced slopes (76.1%). These data indicate that long-term skiing experience correlates with a preference for double boards and pursuit of higher speed and skills.

**Table 4:** Distribution of skiing equipment, skill level, and injury location by skiing experience (*n*, %).

Skiing Experience	Cases (*n*)	Distribution of Skiing Equipment (%)	Distribution of Skill Level (%)	Protective Gear Deficiency (*n*)
<2 years	3032	Double board: 58.08 Single board: 41.92	Beginner: 100.0 Intermediate: 0 Expert: 0	Hip pads: 247 Wrist guards: 70 Knee pads: 610 Helmets: 525
2∼5 years	348	Double board: 63.79 Single board: 36.21	Beginner: 17.53 Intermediate:82.47 Expert: 0	Hip pads: 18 Wrist guards: 163 Knee pads: 4 Helmets: 5
>5 years	890	Double board: 67.3 Single board: 32.7	Beginner: 0 Intermediate:1.35 Expert: 98.65	Hip pads: 72 Wrist guards: 276 Knee pads: 27 Helmets: 15
χ^2^ (*P* value)	-	356.2 (<0.001)	2987.4 (<0.001)	321.5 (<0.001)

Analysis of injury location showed that patients with <2 years of skiing experience mainly suffered from lower extremity injury (38.29%, 1,161 cases, Table 5). Further analysis revealed that more than half of patients with lower extremity injuries do not have knee pads (610 cases, 52.5%, Table 5). Patients with >5 years of skiing experience had the highest upper extremity injury rate (50.8%, 453 cases, Table 5), with 76.1% (678 cases) skiing on advanced slopes. Notably, over 60% of patients with upper extremity injuries do not have wrist guards (276 cases, 60.9%, Table 5). The above analysis reveals significant differences in injury location and protective gaps among patients with different skiing experience levels. Targeted improvements in the use of protective gear and risk management are therefore warranted.

**Table 5:** Distribution of protective gear and injury location by skiing experience (*n*).

Skiing Experience	Distribution of Injury location	Protective Gear Deficiency				
		Helmets	Wrist guards	Knee pads	Hip pads	Unspecified
<2 years	Head and neck	525	0	0	0	47
	Upper extremity	0	70	0	0	744
	Lower extremity	0	0	610	247	304
	Trunk	0	0	0	0	485
2∼5 years	Head and neck	5	0	0	0	25
	Upper extremity	0	163	0	0	57
	Lower extremity	0	0	4	18	34
	Trunk	0	0	0	0	42
>5 years	Head and neck	15	0	0	0	38
	Upper extremity	0	276	0	0	177
	Lower extremity	0	0	27	72	88
	Trunk	0	0	0	0	197

### 3.4 Analysis of injury characteristics based on annual skiing expenditure

There were 629 cases in the high-consumption group (>3000 RMB) and 3,641 cases in the low-consumption group (<3000 RMB). There were significant differences in skiing experience (<2 years, 2∼4 years, >5 years), skill level distribution, and injury location between the high-consumption group and the low-consumption group (all *P* < 0.001, Table 6). The high-consumption group was mainly composed of people with long skiing experience and high skills, with 72.81% (458 cases) having skiing experience >5 years and 447 experts (71.07%). The low-consumption group was mainly composed of people with short skiing experience and low skills, dominated by beginners (84.04%, 3,060 cases). The above data reflect that there is a positive correlation between consumption investment and the accumulation of skiing experience, and high-consumption groups are more likely to improve their skill level through this long-term accumulation process.

**Table 6:** Distribution of skiing experience, skill level and injury location by annual skiing expenditure (*n*, %).

Consumption Stratification	Total Number of Cases	Distribution of Skiing Experience (%)	Distribution of Skill Level (%)	Distribution of Injury location (%)
High consumption (>3000 RMB)	629	<2 years: 0.32 2∼5 years: 26.87 > 5 years: 72.81	Beginner: 5.25 Intermediate: 23.69 Expert: 71.07	Upper extremity: 54.69 Lower extremity: 21.78 Trunk: 18.12 Head and neck: 5.41
Low consumption (<3000 RMB)	3641	<2 years: 83.22 2∼5 years: 4.92 > 5 years: 11.86	Beginner: 84.04 Intermediate: 4.12 Expert: 11.84	Upper extremity: 31.39 Lower extremity: 34.8 Trunk: 16.75 Head and neck: 17.06
χ^2^ (*P* value)	-	1189.5 (<0.001)	1765.2 (<0.001)	372.8 (<0.001)

At the same time, the high-consumption group was mainly affected by upper extremity injury (344 cases, 54.69%), followed by lower extremity injury (137 cases, 21.78%), trunk injury (114 cases, 18.12%), and head and neck injury (34 cases, 5.41%). The low-consumption group was mainly affected by lower extremity injury (1,267 cases, 34.8%), followed by upper extremity injury (1,143 cases, 31.39%), trunk injury (610 cases, 16.75%), and head and neck injury (621 cases, 17.06%).

### 3.5 Multinomial logistic regression analysis

To identify independent predictors of injury location while controlling for multiple variables, multinomial logistic regression was performed with upper extremity injury as the reference category. Results are shown in Table 7. The model was significant (likelihood ratio *χ*^2^ = 10797.3, *P* < 0.001). The overall classification accuracy was 42.6%, with an AUC of 0.684, indicating moderate predictive performance. Age, gender, annual consumption, and skill level were all associated with injury location. With advancing age, the probabilities of head/neck injuries (OR = 1.018, 95%CI: 1.009–1.027), lower extremity injuries (OR = 1.025, 95%CI: 1.017–1.032) and trunk injuries (OR = 1.032, 95%CI: 1.023–1.041) were significantly elevated relative to upper extremity injuries. Compared to females, male skiers had significantly lower odds of head/neck injury (OR = 0.768, 95% CI: 0.631–0.936, *P* = 0.009) and lower extremity injury (OR = 0.639, 95% CI: 0.546–0.749, *P* < 0.001) relative to upper extremity injury. No significant gender difference was observed for trunk injury (*P* = 0.908). Skiers with low annual expenditure (<3000 RMB) had higher odds of head/neck injury compared to upper extremity injury (OR = 1.786, 95% CI: 1.140–2.796, *P* = 0.011) relative to high-expenditure skiers. Compared to expert skiers, beginners had substantially higher odds of head/neck injury (OR = 5.862, 95% CI: 1.774–19.371, *P* = 0.004), lower extremity injury (OR = 4.575, 95% CI: 2.134–9.806, *P* < 0.001), and trunk injury (OR = 2.444, 95% CI: 1.131–5.285, *P* = 0.023).

**Table 7:** Multinomial logistic regression results for injury location (reference: upper extremity injury).

Variable	Comparison (vs. upper extremity)	Adjusted OR	95% CI	*P*-value
Age (per year increase)	Head/neck	1.018	1.009–1.027	<0.001
	Lower extremity	1.025	1.017–1.032	<0.001
	Trunk	1.032	1.023–1.041	<0.001
Gender (male vs. female)	Head/neck	0.768	0.631–0.936	0.009
	Lower extremity	0.639	0.546–0.749	<0.001
	Trunk	1.011	0.838–1.220	0.908
Annual consumption (<3000 vs. ≥3000)	Head/neck	1.786	1.140–2.796	0.011
	Lower extremity	0.981	0.737–1.306	0.896
	Trunk	1.286	0.955–1.733	0.098
Skill level (beginner vs. expert)	Head/neck	5.862	1.774–19.371	0.004
	Lower extremity	4.575	2.134–9.806	<0.001
	Trunk	2.444	1.131–5.285	0.023
Skill level (intermediate vs. expert)	Head/neck	1.619	0.732–3.578	0.234
	Lower extremity	1.056	0.625–1.784	0.839
	Trunk	0.887	0.511–1.538	0.670
Equipment (skis vs. snowboard)	Head/neck	0.942	0.766–1.159	0.573
	Lower extremity	0.921	0.779–1.088	0.335
	Trunk	0.890	0.730–1.085	0.249
Skiing experience (years)	All comparisons	Not significant	—	>0.05

## 4 Discussion

The 18% year-on-year growth in tourist visits to Chongli's ski resorts during the 2024–2025 ski season (reaching 5.2346 million) is a clear indicator of the rapidly growing popularity of skiing in China. However, the inherent high risk of skiing is accompanied by an increasing incidence of skiing-related injuries, which has become a major challenge for the sustainable development of China's snow sports industry. Previous international studies have also confirmed a rising trend in skiing injury rates: Rust et al. reported an increase in skiing injury rates from 206.7 per 100,000 runs to 233.8 per 100,000 runs in a large Western US ski resort, representing a 13.1% increase in absolute injury risk (5). A study on elite alpine skiers in Sweden documented an injury rate of 1.7 injuries per 1,000 skiing hours and 3.11 injuries per 100 skier-months (6). These findings, combined with the results of the present study, highlight the critical need to develop science-based skiing injury prevention strategies tailored to the characteristics of Chinese skiers.

This study's analysis of 4,270 skiing injury cases identified a significant correlation between gender and both injury location and annual skiing expenditure, a finding that provides important insights for gender-differentiated injury prevention. Male skiers were predominantly affected by upper extremity injuries, with 57% of male patients having an annual skiing expenditure exceeding 3000 RMB. This pattern is likely attributable to male skiers' higher propensity for high-risk skiing behaviors (e.g., high-speed jumping, half-pipe skiing) driven by greater risk-taking propensity. During such high-risk maneuvers, accidental falls generate substantial impact forces, and the upper extremities, as the primary weight-bearing support during falls, endure excessive axial loads, which may be associated with injuries such as rotator cuff tears and upper extremity fractures (5, 7). In contrast, female skiers were mainly prone to lower extremity injuries, with the majority having a annual skiing expenditure below 2000 RMB. This gender difference likely arises from multiple factors. Physiologically, the larger Q-angle in females increases knee joint internal rotation torque during skiing, which combined with muscle imbalances or poorly fitted equipment—may elevate lower extremity injury risk (8–10).

Based on these gender-specific injury characteristics, a gender-differentiated skiing injury prevention system is urgently needed in China. For male skiers, the focus should be on resistance training for the upper extremity and rotator cuff muscles (e.g., elastic band external and internal rotation exercises) to enhance joint stability. For female skiers, personalized lower extremity strength training should be implemented to stabilize the lower extremity movement chain (11).

The present study also confirmed a significant correlation between injury location and education level, annual skiing expenditure, skiing experience, and skill level, with a clear pattern: high-expenditure, high-skill skiers were more likely to suffer upper extremity injuries, while low-expenditure, novice skiers had a higher incidence of lower extremity injuries. Notably, high-expenditure expert skiers (72.81% with >5 years of skiing experience) had a high rate of upper extremity injuries, a finding that differs from some previous studies. This unique pattern may be explained by the increased skiing speed and exploration of complex backcountry terrain associated with improved skiing skills, combined with the low rate of wrist guard use (39.1%) in this group. During high-speed skiing, falls (primarily forward or lateral falls) generate enormous impact forces, and the lack of wrist protection is associated with upper extremity injuries. Although the relatively young age of the study population (58.78% 20–35 years) may be a contributing factor, the limited protective effect of current conventional protective gear against high-impact forces during high-speed skiing is a more critical issue that needs to be addressed (12).

In contrast, the high incidence of lower extremity injuries in the low-expenditure group (predominantly novice alpine skiers) is consistent with the findings of Zachary et al., who reported that alpine ski-related injuries are most commonly anterior cruciate ligament (ACL) injuries and tibial fractures (9). Novice skiers lack the ability to maintain proper center of gravity during skiing, leading to knee joint torsion and subsequent injuries. Csapo et al. demonstrated that cold temperatures during skiing reduce the recruitment rate of knee flexor muscles, thereby impairing the body's ability to resist shear forces and hyperextension, increasing the risk of ACL injuries (13, 14). Ishimaru et al. also found that the most common lower extremity injury types in skiers are lacerations and contusions (followed by sprains), accounting for 61.8% of all lower extremity injuries; male skiers are more prone to lacerations and fractures, while female skiers have a higher rate of contusions and posterior lower extremity injuries (15).

Based on the analysis of data from skiing injury patients admitted to the Chongli Campus during the 2024–2025 ski season, this study revealed the inherent patterns of skiing injuries. The prevention of skiing injuries is particularly important. Different preventive measures are recommended for skiers of different genders and skiing experience levels. Skiers can strengthen their athletic abilities in a targeted manner to reduce the risk of injury. In addition, skiers should actively wear suitable skiing protective gear such as helmets and knee pads.

Regarding the multinomial logistic regression model, the overall classification accuracy was only 42.6% with an AUC of 0.684, indicating limited predictive performance. This may be due to the exclusion of potentially important variables (e.g., detailed injury mechanisms, terrain type, weather conditions) and the inherent heterogeneity of skiing injuries. Future studies should incorporate more granular exposure data to improve model discriminative ability.

A recent five-year retrospective study by Subaşı et al. (16) investigated isolated extremity injuries among 304 recreational skiers and snowboarders treated at a tertiary trauma center in Turkey (16). In their cohort, 53.2% of patients sustained lower extremity injuries and 46.8% upper extremity injuries, which is comparable to our findings (upper extremity 34.8%, lower extremity 32.9%; our full cohort also included trunk and head/neck injuries). Notably, their study excluded injuries to the head, face, thorax, and back, whereas our study included all body regions, providing a more complete picture of skiing-related trauma. Despite these differences, the consistency in extremity injury patterns across two geographically distinct populations (Turkey and China) supports the external validity of our findings and suggests that similar preventive strategies may be effective internationally.

This study has certain limitations. The data source of this study is relatively limited. Additionally, key variables such as skill level, skiing experience, and protective gear use were self-reported and lacked external validation, which may introduce recall and classification bias. The data were only obtained from the Chongli Campus and cannot fully represent the injury status of all skiers in this ski season. Some skiing enthusiasts suffered minor injuries and did not seek medical treatment in the hospital. This may cause a certain bias in the evaluation of the injury status in this study. Moreover, this study only included data from a single ski season. The results are difficult to fully reflect the long-term trend of skiing injuries. This study mainly conducted statistical analysis around injury locations and did not further explore the specific occurrence mechanisms of various injuries. The relevant conclusions still need to be further verified through subsequent interventional studies. Future studies should comprehensively collect specific injury diagnoses and causes, and further analyze the underlying mechanisms and patterns of injuries.

## 5 Conclusions

This retrospective study analyzed 4,270 skiing injury cases in Chongli District during the 2024–2025 ski season. The findings confirm that skiing injury occurrence is associated with gender, age, annual skiing expenditure, skiing experience, and skill level, with distinct injury patterns observed across different skier subgroups. Multinomial logistic regression identified age, male gender (protective for head/neck and lower extremity injuries relative to upper extremity), low annual consumption (associated with higher odds of head/neck injury), and beginner skill level (associated with higher odds of lower extremity and trunk injuries) as independent predictors. This study fills a gap in localized epidemiological research on skiing injuries in Chongli District and provides empirical data for developing targeted prevention strategies in China.

## Data Availability

The original contributions presented in the study are included in the article/Supplementary Material, further inquiries can be directed to the corresponding author.

## References

[1] Chen N Yang Y Jiang Y Ao Y. Injury patterns in a large-scale ski resort in the host city of 2022 winter Olympic games: a retrospective cross-sectional study. BMJ Open. (2020) 10:e037834. 10.1136/bmjopen-2020-037834 33234619 PMC7684805

[2] Ma X Li JY Andd SG Ao YF Yang YP. Comparison and analysis of skiing injuries at ski resorts in chongli, China and Japan. Chin J Traumatol. (2023) 26:63–7. 10.1016/j.cjtee.2022.08.002 36180308 PMC10071314

[3] Asadollahi S Bucknill A. Acute medial clavicle fracture in adults: a systematic review of demographics, clinical features and treatment outcomes in 220 patients. J Orthop Traumatol. (2019) 20:24. 10.1186/s10195-019-0533-3 31254115 PMC6598891

[4] Jacobsson J Timpka T. Classification of prevention in sports medicine and epidemiology. Sports Med. (2015) 45:1483–7. 10.1007/s40279-015-0368-x 26245875

[5] Rust DA Gilmore CJ Treme G. Injury patterns at a large western United States ski resort with and without snowboarders: the taos experience. Am J Sports Med. (2013) 41:652–6. 10.1177/0363546512472045 23324432

[6] Tarka MC Davey A Lonza GC O'Brien CM Delaney JP Endres NK. Alpine ski racing injuries. Sports Health. (2019) 11:265–71. 10.1177/1941738119825842 30689522 PMC6537318

[7] Koehle MS Lloyd-Smith R Taunton JE. Alpine ski injuries and their prevention. Sports Med. (2002) 32:785–93. 10.2165/00007256-200232120-00003 12238941

[8] Sporri J Kroll J Gilgien M Muller E. How to prevent injuries in alpine ski racing: what do we know and where do we go from here? Sports Med. (2017) 47:599–614. 10.1007/s40279-016-0601-2 27480763 PMC5357247

[9] Telgheder ZL Kistler BJ. Ski and snowboard-related orthopedic injuries. Orthop Clin North Am. (2020) 51:461–9. 10.1016/j.ocl.2020.06.004 32950215

[10] Promsri A Longo A Haid T Doix AM Federolf P. Leg dominance as a risk factor for lower-limb injuries in downhill skiers. A pilot study into possible mechanisms. Int J Environ Res Public Health. (2019) 16:3399. 10.3390/ijerph16183399 31540226 PMC6765833

[11] Hebert-Losier K Holmberg HC. What are the exercise-based injury prevention recommendations for recreational alpine skiing and snowboarding? A systematic review. Sports Med. (2013) 43:355–66. 10.1007/s40279-013-0032-2 23463392

[12] Provance AJ Daoud AK Tagawa A Rhodes J. Pediatric and adolescent injury in skiing. Res Sports Med. (2018) 26:150–65. 10.1080/15438627.2018.1438282 30431354

[13] Davey A Endres NK Johnson RJ Shealy JE. Alpine skiing injuries. Sports Health. (2019) 11:18–26. 10.1177/1941738118813051 30782106 PMC6299353

[14] Csapo R Folie R Hosp S Hasler M Nachbauer W. Why do we suffer more ACL injuries in the cold? A pilot study into potential risk factors. Phys Ther Sport. (2017) 23:14–21. 10.1016/j.ptsp.2016.07.004 27639576

[15] Ishimaru D Ogawa H Sumi H Sumi Y Shimizu K. Lower extremity injuries in snowboarding. J Trauma. (2011) 70:E48–52. 10.1097/TA.0b013e31820ca025 21610335

[16] Subasi IO Gur V. Recreational skiing and snowboarding-related extremity injuries: a five-year tertiary trauma center cohort. Cureus. (2023) 15:e42688. 10.7759/cureus.42688 37649954 PMC10464917

